# The m^6^A Dynamics of *Profilin* in Neurogenesis

**DOI:** 10.3389/fgene.2019.00987

**Published:** 2019-11-12

**Authors:** Antonio L. Rockwell, Cintia F. Hongay

**Affiliations:** Department of Biology, Clarkson University, Potsdam, NY, United States

**Keywords:** m^6^A effector, profiling, IME4, alternative splicing, RNA processing alterations

## Abstract

Our understanding of the biological role of *N* ^6^-methyladenosine (m^6^A), a ubiquitous non-editing RNA modification, has increased greatly since 2011. More recently, work from several labs revealed that m^6^A methylation regulates several aspects of mRNA metabolism. The “writer” protein METTL3, known as MT-A70 in humans, *Dm*Ime4 in flies, and MTA in plants, has the catalytic site of the METTL3/14/16 subunit of the methyltransferase complex that includes many other proteins. METTL3 is evolutionarily conserved and essential for development in multicellular organisms. However, until recently, no study has been able to provide a mechanism that explains the essentiality of METTL3. The addition of m^6^A to gene transcripts has been compared with the epigenetic code of histone modifications because of its effects on gene expression and its reversibility, giving birth to the field of *epitranscriptomics*, the study of the biological role of this and similar RNA modifications. Here, we focus on METTL3 and its likely conserved role in profilin regulation in neurogenesis. However, this and many other subunits of the methyltransferase complex are starting to be identified in several developmental processes and diseases. A recent plethora of studies about the biological role of METTL3 and other components of the methyltransferase complex that erase (FTO) or recognize (YTH proteins) this modification on transcripts revealed that this RNA modification plays a variety of roles in many biological processes like neurogenesis. Our work in *Drosophila* shows that the ancient and evolutionarily conserved gene *profilin* (*chic* in *Drosophila*) is a target of the m^6^A writer. Here, we discuss the implications of our study in *Drosophila* and how it unveils a conserved mechanism in support of the essential function of METTL3 in metazoan development. *Profilin* (*chic*) is an essential gene of ancient evolutionary origins, present in sponges (Porifera), the oldest still extant metazoan phylum of the common metazoan ancestor Urmetazoa. We propose that the relationship between *profilin* and METTL3 is conserved in metazoans and it provides insights into possible regulatory roles of m^6^A modification of *profilin* transcripts in processes such as neurogenesis.

## Introduction

Dozens of RNA modifications have been identified to date. However, the function of these chemical modifications in gene transcripts remains largely elusive (Hsu et al., 2017). One of the best studied is the *N* ^6^-methyladenosine (m^6^A) modification. The m^6^A modification is the most abundant non-editing internal modification of eukaryotic mRNA (Yue et al., 2015). The m^6^A modification is added by METTL3/14/16 writer proteins, which are members of the methyltransferase complex (Liu and Pan 2016). METTL3 and its homologs, IME4 (yeast), *Dm* Ime4 (*Drosophila*), MTA (*Arabidopsis*), and MT-A70 (human), have the catalytic structure for Ado-Met binding and subsequent methylation of adenosine residues by the methyltransferase complex (Clancy et al, 2002), while the other components of the complex aid in the recognition of the RNA consensus sequence that contains the adenosine to be methylated, which is frequently found in the context of hairpin configurations (Wang et al., 2016). For simplicity, we will call all these homologs Mettl3 throughout this article. In addition to the Mettl3/14/16 subunit, other proteins are an integral part of the methyltransferase complex. Among these other proteins are Zf3h13, WTAP, Virilizer, Spenito, Hakai, KIAA1429, and RBM15. It is not yet understood whether all the components of the methyltransferase complex are present in every cell of every organism and in every developmental context or whether the composition of the methyltransferase complex is developmentally regulated and varies depending on cell type, organism, and developmental stage. However, the Mettl3 proteins are constant components of this complex and evolutionarily conserved throughout metazoans. The m^6^A mark on RNA is recognized by readers such as YTH proteins, and it is reversible, as it can be removed by eraser proteins such as FTO (Cao et al., 2016; Liu and Pan 2016; Roundtree et al., 2017; Spychala and Ruther, 2019). m^6^A can potentially regulate several aspects of RNA metabolism depending on where this mark resides on the RNA molecule (Liu and Pan 2016). The location of the modification on the transcript is thought to regulate specific aspects of RNA metabolism (Liu and Pan 2016; Covelo-Molares et al., 2018). For example, the modification in the 5′UTR results in translation regulation, while the modification in the 3′UTR regulates RNA stability (Wang et al., 2014; Liu and Pan, 2016).

Errors in the incorporation of this modification onto RNA have detrimental consequences in biological processes, such as angiogenesis, stem cell maintenance, gametogenesis, and development, and can cause cancers (Niu et al., 2013; Miao et al, 2019). Recent investigations suggest brain development is another process that relies on this modification (Angelova et al., 2018; Shi et al., 2018; Spychala and Ruther, 2019). Given the function of the brain in learning and memory, two processes that rely on neuronal plasticity, it is not surprising that the epitranscriptome is the key regulatory layer of gene regulation in brain function. Proteins involved in processing the m^6^A modification are being assigned important roles in neurological development (Shi et al., 2018). Mettl3 and other components of the methyltransferase complex have strong localization in eukaryotic neuronal tissue (Angelova et al., 2018). For example, YTH readers are expressed at high levels in *Drosophila* and murine brain tissue (Hartmann et al., 1999; Lence et al., 2016). An additional example is the FTO eraser protein, which is expressed at high levels in human and murine hypothalamus (McTaggart et al., 2011; Angelova et al., 2018; Spychala and Ruther, 2019). Besides localization, additional studies suggest a more direct connection of the methyltransferase complex to neurological disorders. For instance, the Zfh13 appears to be a marker for schizophrenia, a neurological disorder (Oldmeadow et al., 2014). Zfh13 was shown to have a single-nucleotide polymorphism (SNP) mutation in schizophrenia patients using genome-wide screening. Interestingly, one of the best characterized members of the methyltransferase complex, the writer protein Mettl3, has also been shown to be required for brain development and function (Visvanathan et al., 2018; Wang et al., 2018).

Mettl3, the catalytic subunit of the methyltransferase complex (Clancy et al., 2002; Yang et al., 2018), is encoded by an essential gene in many eukaryotic organisms (Zhong et al., 2008; Hongay and Orr-Weaver 2011; Guela et al., 2015; Rockwell et al., 2019). Since *mettl3* is essential, manipulating the gene to determine its function *in vivo* has been challenging (Rockwell et al., 2019). Therefore, most studies are performed *ex vivo*, using cell and tissue cultures or *in vitro* with partial biochemical reconstructions of the complex and its substrates. Consequently, the mechanism of Mettl3 in processes such as brain development *in vivo* and in the context of a whole organism is not completely understood. To circumvent this challenge, we have manipulated the expression levels of *mettl3 via* RNAi to bypass its essential requirement for viability and observed the consequences of *mettl3* ablation in the non-essential developmental context of spermatogenesis (Rockwell et al., 2019). Using the aforementioned experimental approach, we have found that *profilin* (*chic* in *Drosophila*) transcript and protein levels are affected by reduction of Mettl3. We postulate that Mettl3’s regulation of *profilin* is conserved in other metazoans and developmental scenarios. Given that profilin is an ancient, evolutionarily conserved, and essential protein required for metazoan development (Müller, 2003; Müller and Müller, 2003), the regulation of *profilin* by Mettl3 can shed light on Mettl3’s role in evolutionarily conserved and essential biological processes that require profilin function such as brain development.

## Mettl3’s Role in Profilin Splicing and Processing

In most eukaryotes, multiple variants of a protein are generated by alternative splicing of the transcripts that are encoded by a gene. Alternative splicing is developmentally regulated, and genes can generate specific protein variants (spliceoforms) according to the developmental stage of the organism and cell type, tissue type, and organ type. *Profilin* genes can generate different spliceoforms (Witke 2004). The spliceoforms of *profilin* are often tissue specific (Witke 2004). Unfortunately, the mechanism that determines which spliceoform is generated in certain cells but not others is not completely understood. Our soon to be published studies in *Drosophila* show that Mettl3 is required for *profilin* (*chic*) splicing. Controlled depletion of Mettl3 to bypass its essential function using the Gal4–UAS system resulted in accumulation of unspliced *chic* transcript. Ours is the first study that postulates a possible mechanism for *profilin* splicing. Although our work shows this interaction in *Drosophila*’s spermatogenesis, for this review, we use sequencing data publicly available in the genome browser (described in Kent et al., 2002) to identify Mettl3’s consensus binding sites *in silico* in mammalian *profilin* mRNAs to propose that Mettl3 may interact with *profilin* transcripts in other metazoans, specifically mammals.

**Figure 1:**
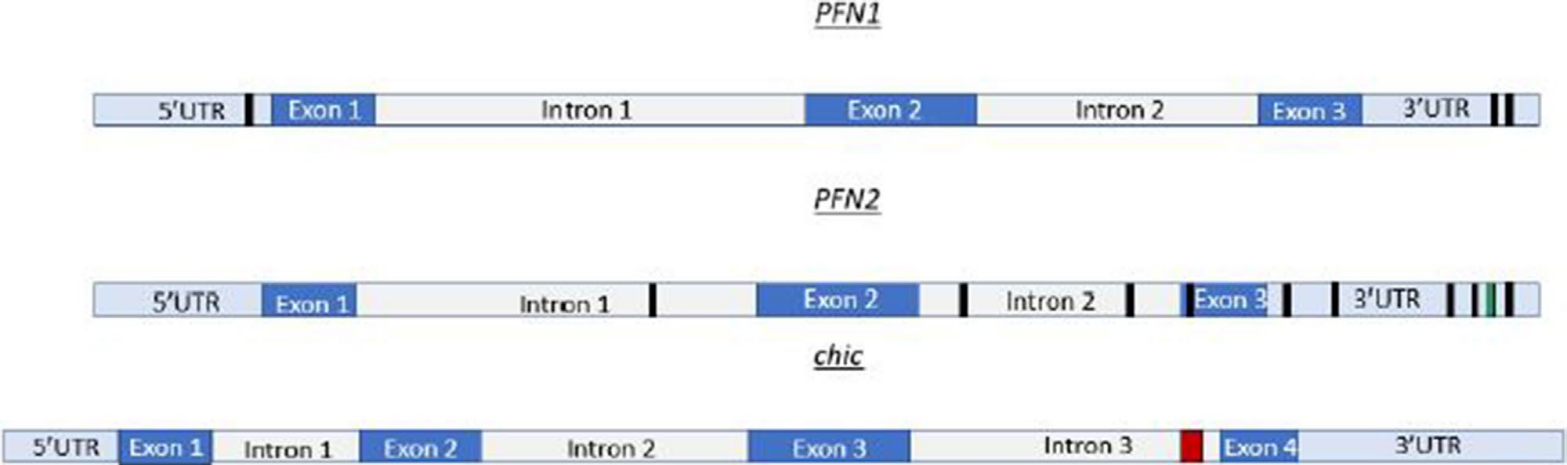
*Profilin* transcripts have multiple Mettl3 binding sites. *PFN1* mRNA, depicted in cartoon form at the top, has Mettl3 binding sites (AAACC) depicted by black boxes in the 3′UTR and 5′UTR. *PFN2* mRNA represented as the transcript in the middle of this figure, has Mettl3 binding sites (AAACA) in the 3′UTR, exon 3, intron 1, and intron 2. *PFN2* mRNA has additional binding sites in 3′UTR represented by green box (UGUGGACU). *chic* (*Drosophila* profilin), depicted as the bottom cartoon in this figure, has a cluster of METTL3 binding sites (GTTCTTATTTCTCCGCCGCTGACGGTG) in intron 3 represented by red box. This cluster, when run through appropriate algorithms, can generate hairpins for complex recognition.

The *in silico* analysis of *profilin* transcripts reveals multiple Mettl3 binding sites. For example, mRNA sequencing data in humans show multiple Mettl3 bindings sites on *profilin 1* (*PFN1*) and *profilin 2* (*PFN2*) (**Figure 1**). In *Drosophila*, there are a cluster of Mettl3 binding sites in the *chic* (*profilin*) transcript (**Figure 1**). *PFN1* is homologous to *chic* in *Drosophila*. For our *in silico* inquiry, we used consensus sequences known to have high affinity for Mettl3 binding. The sequences used in **Figure 1** are AAACC (*PFN1*), AAACA and UGUGGACU (*PFN2*), and GTTCTTATTTCTCCGCCGCTGA CGGTG (*chic*). These binding sites are localized on different portions of the transcript. Some of these binding sites are in the 5′UTR and 3′UTR, while others are in the exon and intron regions. *PFN2* has many Mettl3 binding sites throughout the transcript, which include the 3′UTR, exon 3, intron 1, and intron 2. There are two known spliceoforms of *PFN2* (*PFN2a* and *PFN2b*). We propose that recognition and use of these sites by Mettl3 aided by the other components of the methyltransferase complex may vary in different developmental contexts to generate the spliceoforms needed to be synthesized. Conversely, *PFN1* only has a few Mettl3 binding sites, two in the 3′UTR and one in the 5′UTR. Taken together, our studies in *Drosophila* and the *in silico* identification of Mettl3 binding sites on profilin transcripts (**Figure 1**) suggest an evolutionarily conserved relationship between the methyltransferase complex and the regulation of the expression of this ancient gene. Interestingly, a similar Mettl3 recognition site is present in PFY1, the profilin gene in budding yeast.

### The Methyltransferase Complex in Neurogenesis

Mettl3 plays a role in neurogenesis in mammals and *Drosophila*. *Mettl3* is essential in mouse, as a complete deletion of this gene results in early embryonic arrest (Geula et al., 2015). In mouse, m^6^A methylation regulates cortical neurogenesis (Yoon et al., 2017). Depletion of Mettl3 and/or Mettl14 in murine results in decreased m^6^A levels (Yoon et al., 2017). Knockdown of *Mettl3* in mouse using an *in vivo* short hairpin RNA shRNA technique results in an increase in the length of the cell cycle and defects in maintenance of radial glial cells (Yoon et al., 2017). *Mettl14* conditional knockout using the *Nestin–Cre* system in mouse embryos also results in a prolonged cell cycle and longer cortical neurogenesis (Yoon et al., 2017). Besides the cortical neurogenesis investigation, other studies also found defects in m^6^A modification impacted normal neuronal capabilities. For example, *Mettl14* deletion in two striatum subgroups resulted in decrease in m^6^A levels (Koranda et al., 2018). This decrease in m^6^A levels coincided with increase neuronal excitability and impaired striatum function, likely due to mRNA levels encoding synapse specific proteins being downregulation (Koranda et al., 2018). Another study that examined the impact of m^6^A in synapses found this modification is likely required for proper synapse function (Merkurjev et al., 2018). Additionally, depletion of METTL3 using *Lox-Cre* in mouse results in an altered epitranscriptome and abnormal behavioral defects (Engel et al., 2018). The altered epitranscriptome is likely affecting proteins needed for proper brain function such as profilin. Similarly, in *Drosophila*, depletion of the *Mettl3* results in abnormal behavioral defects, a flightless phenotype, and aberrant neuromuscular junctions (NMJs) (Haussmann et al., 2016; Lence et al., 2016). The locomotion and flightless defects are likely related and possibly due to defects in NMJ characterized by a “held-out wing” phenotype (Haussmann et al., 2016; Lence et al., 2016), the basis of which has yet to be determined. Here, we propose that the common thread of these neurological defects, which have been described but not molecularly analyzed, may be profilin.

### Profilin Molecular Function and Neuronal Expression Pattern

**Figure 2:**
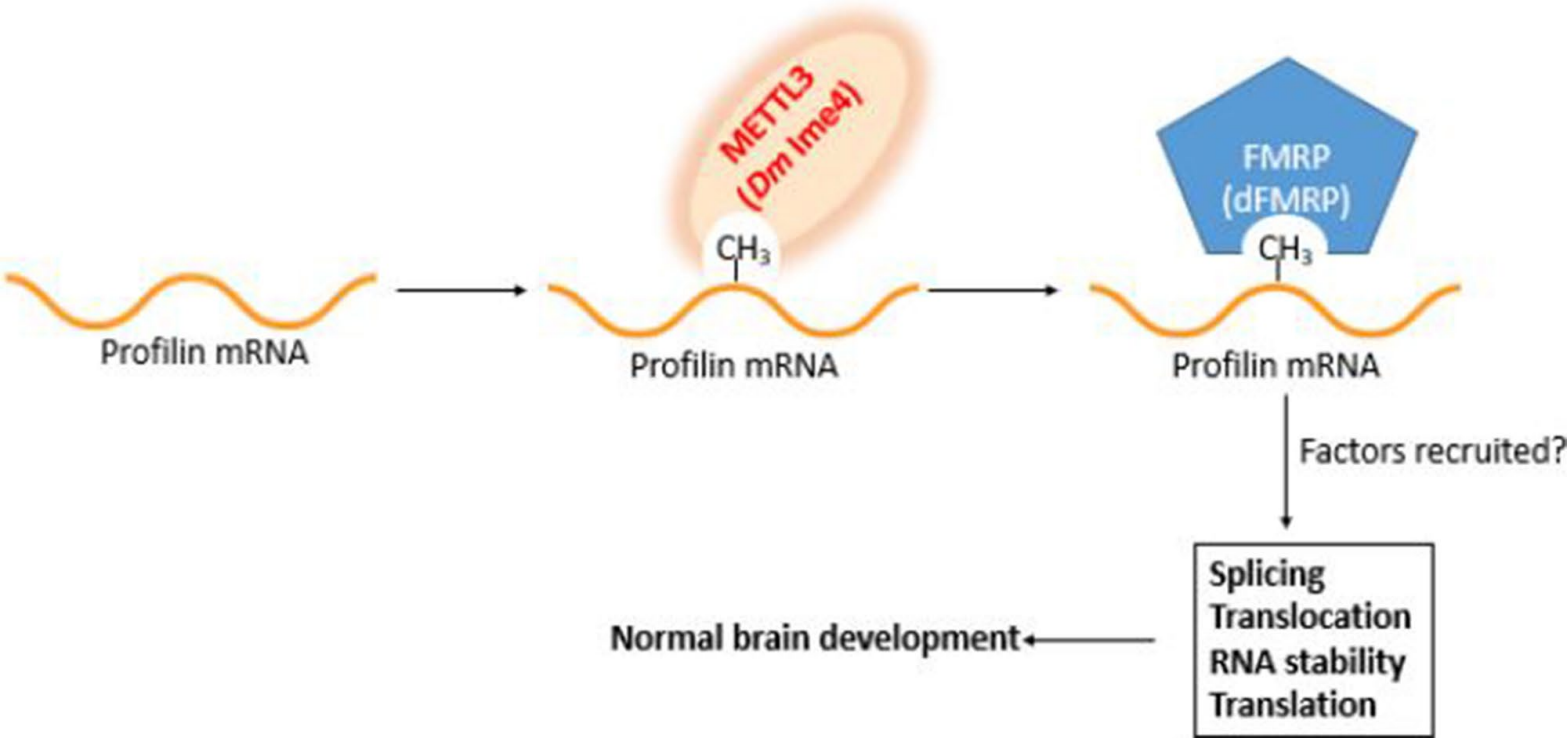
Working model for profilin regulation in the brain. *Profilin* pre-mRNA is methylated by Mettl3 upon binding site recognition aided by Mettl14/16 and other components of the methyltransferase complex. The m^6^A mark is recognized by the m^6^A reader FMRP (dFMRP). The marking and its recognition are required for the recruitment of splicing and processing factors. Failure to properly mark and recognize *profilin* pre-mRNA has deleterious consequences in brain development.

*Profilin* is an essential gene in development (Witke 2004; Geula et al., 2015). Profilin has several proposed roles in the cell (**Figure 2**). The best characterized function is as actin binding protein required for F-actin polymerization, a housekeeping role, and as such, mammalian *PFN1* is ubiquitously expressed. Conversely, *profilin 2a* (*PFN2a* and *PFN2b*) is not a housekeeping gene, and it is only expressed in the central nervous system (CNS), primarily in brain tissue (Witke 2004). PFN1 has a strong affinity for actin and poly--proline, and it typically binds ligands that range from 45 to 190 kDa. PFN2a is like PFN1, as it also has a high affinity for actin and poly--proline (Dinardo et al., 2000). Interestingly, PFN2b does not bind actin and has a lower affinity for poly--proline (Dinardo et al., 2000). PFN2b has a strong affinity for tubulin. In *Drosophila*, there are several annotated profilin spliceoforms that generate from *chic* (Kent et al., 2002). However, only ovary-specific and constitutive spliceoforms have been identified (Verheyen et al., 1994). There are still spliceoforms that need to be characterized. It is possible that epitranscriptome modifications are required for the processing of these spliceoforms in a tissue-specific manner, and future studies are needed to elucidate the underlying mechanisms.

### PFN1 in Neurogenesis

Many proteins are required to regulate neurogenesis. PFN1, a protein that is vital for a glial cell’s function, is one of these. In Schwann cells (SCs), PFN1 is required for lamellipodia formation, a key requirement for myelination (Montani et al., 2014). It was found that when shRNA was used to knockdown *PFN1* in SC cultures, the knockdown resulted in reduced formation of axial and radial lamellipodia (Montani et al., 2014). Myelination is important for the propagation of action potentials and normal functioning nervous system. PFN1 regulates cytoskeletal remodeling, which is necessary for lamellipodia formation. PFN1 may play other important roles in neurons. PFN1 is present at the neuronal synapse and colocalizes with the synapse protein synaptophysin (Neuhoff et al., 2005). This suggests a role of profilin and ultimately actin in synapse function. The regulation of PFN1 in the brain is critical for normal neurological function, as defects in PFN1 regulation can result in mental abnormalities (Michaelsen-Preussea et al., 2006). Therefore, the identification of proteins that regulate PFN1 will have important implications in testing and perhaps devising treatments for mental abnormalities. One such protein linked to PFN1 regulation in the brain is the fragile X mental retardation protein (FMRP) (Michaelsen-Preussea et al., 2006).

In mouse, FMRP binds directly to *PFN1* through a novel RNA binding motif (Michaelsen-Preussea et al., 2006). It is unknown whether the motif recognized by FMRP is a target of the methyltransferase complex. The relationship between *PFN1* and is conserved in *Drosophila* as well. As mentioned previously, PFN1 is homologous to profilin in *Drosophila*. In *Drosophila*, FMRP is called dFMRP, and it has been shown to bind to the *chic* transcript (Reeve et al., 2005). The interaction between dFMRP and *chic* was examined in an immunoprecipitation experiment using an anti-dFMRP antibody. dFMRP is needed for proper neuronal development and circadian rhythm (Reeve et al., 2005). In *Drosophila*, dFMRP mutants have defects in cytoskeleton dynamics, which eventually manifest into behavioral abnormalities (Reeve et al., 2005). In humans and mouse, defective FMRP results in fragile X syndrome and other intellectual disorders (IDs) (Michaelsen-Preussea et al., 2006). *FMRP* knockout mice have low levels of PFN1, which affect translation in dendrites (Michaelsen-Preussea et al., 2006). FMRP has been shown to be critical for normal brain development (Angelova et al., 2018). Interestingly, studies suggest that FMRP is a m^6^A reader (Angelova et al., 2018). Future studies are needed to investigate the relationship between FMRP and Mettl3, as they may reveal important features of the subunit composition of the methyltransferase complex as a function of cell type specificity. *Ex vivo* and *in vitro*, all m^6^A readers can recognize and bind to transcripts that contain the methylation of *N* ^6^ residues on adenosine (Cao et al., 2016; Liu and Pan 2016; Yang et al., 2018). However, it is unknown whether this is true *in vivo*. We propose that m^6^A recognition is much more specific *in vivo* and depends on when and where m^6^A is added to the target transcript by Mettl3.

### PFN2 in Neurogenesis

PFN2 is expressed specifically in brains consistent with PFN2’s requirement for neurogenesis. In mouse, PFN2 is needed for axon and dendritic processes (Witke et al., 1998). PFN2a is required for neuritogenesis, the first step of neuronal differentiation, a process critical for neurogenesis in the developing brain (Da Silva et al., 2003). PFN2a regulates neuritogenesis by regulating actin stability as determined using cultured mouse hippocampal neurons. Reduction of PFN2a levels in hippocampal neurons using a morpholino techniques resulted in neurite branching overgrowth, which is atypical in neuritogenesis. The mechanism that ensures specific expression of the *PFN2a* isoform in brain is not understood. Based on our *in silico* analysis (**Figure 1**), we propose that Mettl3 is required for m^6^A marking of *PFN2* to generate specifically the *PFN2a* spliceoform in brain tissue during neurogenesis.

## Conclusion

It is now widely accepted that modifications to RNA have vital roles in regulating normal cell function. The current work on the m^6^A modification further supports the importance of RNA modifications in processes such as neurological development. This makes finding targets of the methyltransferase complex that contribute to neurological development significant for advancing translational venues in human health. Here, we describe the *profilin* transcript as one target that can be methylated and recognized by the methyltransferase complex. Our *in silico* identification of Mettl3 consensus binding sites along the *profilin* transcripts show that these sites are located on different parts of the mRNA. Future studies will be needed to discern which of these sites are bound or targeted for methylation by the methyltransferase complex. It is possible that some of the sites are not recognized or that they are only recognized in a cell-type-specific manner, while other sites may act constitutively. Although the composition of the methyltransferase complex has been elucidated, it is still unknown whether the composition of this multi-subunit enzymatic complex is the same in all cells or whether it varies according to cell type, organism, and/or developmental stage. Arguing against ubiquitous marking and recognition of adenosines that are found within the consensus sequence in pre-mRNA is the fact that not every adenosine residue gets methylated. The consensus site needs to be sterically presented in hairpin configurations, and there is steric hindrance provided by the catalytic pockets formed in the Mettl3/14/16 subunits of the methyltransferase complex. Other recognition restrictions can occur by the positioning of other components of the complex. Studying recognition, methylation, and complex composition in the context of profilin m^6^A marking for mRNA processing can prove a valuable strategy to answer outstanding questions in the field of epitranscriptomics. Mettl3 was the first component of the methyltransferase complex to be identified, and it has been linked to several debilitating neurological conditions that are challenging to diagnose and treat. Further investigation of Mettl3 could open new therapeutic venues and help treat certain neurological conditions. Although this review has focused on *profilin* as a target, other transcripts remain to be identified and studied. For example, in glioblastoma cells, *ADAM19* is an oncogene that promotes tumor progression. Mettl3 normally methylates *ADAM19* mRNA, resulting in the degradation of *ADAM19* required for tumor suppression (Deng et al., 2018). In glioblastoma cells, *Mettl3* is downregulated, leading to upregulation of *ADAM19*. It is undeniable that, thanks to the recent technological advances, the field of epitranscriptomics is in an accelerated phase of discovery. The role of m^6^A mRNA as a gene expression regulatory mark will enrich our understanding of gene expression regulation the same way the discovery of the histone modifications did over 30 years ago.

## Author Contributions

ALR wrote the fiirst draft and CFH (corresponding author) edited and prepared the review submitted for publication.

## Conflict of Interest

The authors declare that the research was conducted in the absence of any commercial or financial relationships that could be construed as a potential conflict of interest.
